# Pharmacokinetics of Repeated Sodium Salicylate Administration to Laying Hens: Evidence for Time Dependent Increase in Drug Elimination from Plasma and Eggs

**DOI:** 10.1371/journal.pone.0123526

**Published:** 2015-04-20

**Authors:** Błażej Poźniak, Tomasz Grabowski, Karolina Motykiewicz-Pers, Kamila Bobrek, Lech Rak, Katarzyna Bobusia, Andrzej Gaweł, Marcin Świtała

**Affiliations:** 1 Department of Biochemistry, Pharmacology and Toxicology, Faculty of Veterinary Medicine, Wrocław University of Environmental and Life Sciences, Wrocław, Poland; 2 Polpharma Biologics, Gdańsk, Poland; 3 Department of Epizootiology and Clinic of Birds and Exotic Animals, Faculty of Veterinary Medicine, Wrocław University of Environmental and Life Sciences, Wrocław, Poland; 4 Department of Food Hygiene and Consumer Health Protection, Faculty of Veterinary Medicine, Wrocław University of Environmental and Life Sciences, Wrocław, Poland; Public Health Research Institute at RBHS, UNITED STATES

## Abstract

Salicylates were the first non-steroid anti-inflammatory drugs (NSAIDs) to be used in any species and are still widely used in humans and livestock. However, the data on their pharmacokinetics in animals is limited, especially after repeated administration. Evidence exist that in chickens (*Gallus gallus*) salicylate (SA) may induce its own elimination. The aim of this study was to investigate salicylate pharmacokinetics and egg residues during repeated administration of sodium salicylate (SS) to laying hens. Pharmacokinetics of SA was assessed during 14 d oral administration of SS at daily doses of 50 mg/kg and 200 mg/kg body weight to laying hens. On the 1^st^, 7^th^ and 14^th^ d a 24 h-long pharmacokinetic study was carried out, whereas eggs were collected daily. Salicylate concentrations in plasma and eggs were determined using high-performance liquid chromatography with ultraviolet detection and pharmacokinetic variables were calculated using a non-compartmental model. Mean residence time (MRT), minimal plasma concentration (C_min_, C_16h_) and elimination half-life (T_1/2el_) of SA showed gradual decrease in layers administered with a lower dose. Total body clearance (Cl_B_) increased. Layers administered with the higher dose showed a decrease only in the T_1/2el_. In the low dose group, SA was found only in the egg white and was low throughout the experiment. Egg whites from the higher dose group showed initially high SA levels which significantly decreased during the experiment. Yolk SA levels were lower and showed longer periods of accumulation and elimination. Repeated administration of SS induces SA elimination, although this effect may differ depending on the dose and production type of a chicken. Decreased plasma drug concentration may have clinical implications during prolonged SS treatment.

## Introduction

Salicylates were the first non-steroid anti-inflammatory drugs (NSAIDs) to be used in any species. In poultry medicine, those used most often are acetylsalicylic acid (ASA) and sodium salicylate (SS) because of their well-known anti-inflammatory and analgesic properties [1]. Other uses are for treating heat stress, ascites, locomotor disorders, stimulation of egg production and improving eggshell thickness, as well as for respiratory and digestive problems [2–6]. Under clinical conditions, salicylates are administered for several days, even up to a few weeks. Up to date, pharmacokinetics of SS in poultry was investigated only after single administration. Baert and De Backer [1,7] compared the disposition of SS after intravenous (*i*.*v*.) administration to chickens, turkeys, ducks, pigeons and ostriches. Mohammad *et al*. [8] investigated the pharmacokinetics of salicylate (SA) after single intraperitoneal injection of ASA in chickens, and recently, our group compared the pharmacokinetics of ASA and SS after single *i*.*v*. and oral administration in chickens and turkeys [9]. There is, however, evidence that during repeated administration of SS, the pharmacokinetics of SA in chickens undergoes significant changes that were never before observed in other species. In our earlier study, we have observed that the trough concentration of SA in chickens during two-week daily administration of ASA or SS had gradually decreased [10]. Since minimal plasma SA concentration was the only pharmacokinetic parameter investigated in this study, it seemed necessary to provide a more complete pharmacokinetic description of this effect.

The aim of this study was to investigate the changes in SA pharmacokinetics during repeated administration of SS to hens. The study was carried out on laying hens to assess whether the proposed mechanism of metabolic induction applies to the presence of SA in eggs.

## Materials and Methods

### Animals and experimental protocol

The experiment was carried out on 16 White Leghorn laying hens (40 weeks old) weighing 2.74 ± 0.30 kg. The birds were kept individually in cages (floor surface: 1800 cm^2^, height: 45 cm) in the Animal House of the Veterinary Faculty in Wrocław (lighting 14 h per day, temperature of 25±2°C, optimal ventilation) and provided with full access to commercial food and water ad libitum. The experiment was approved by the II Local Ethics Committee for Animal Experiments in Wrocław (permit number 77/2012). All procedures involving animals were performed in accordance with national and international laws and policies. All efforts were made to minimize animals’ suffering and to reduce the number of animals used. Layers were randomly divided into two groups with 8 individuals each. Sodium salicylate (of pharmaceutical grade, kindly provided by VETOS-FARMA, Bielawa, Poland) was administered daily as water solution for 14 d at a dose of 50 mg/kg or 200 mg/kg orally via a soft tube into the crop in an appropriate volume (1 ml/kg). Fresh SS solutions were prepared daily prior to administration. On the 1^st^, 7^th^ and 14^th^ d a 24 h-long pharmacokinetic study was carried out. Each time, 1 ml of blood was sampled into heparinised 2 ml syringes (Polfa, Lublin, Poland) with 23G injection needles using metatarsal venipuncture at the following time points: 0 (immediately before the drug administration), 0.5, 1, 2, 4, 8, 16 and 24 h after drug administration. Blood was centrifuged (1700 g, 15 min) and plasma was collected, and stored at -70°C until assay for SA concentration. Eggs from each hen were being collected on a daily basis. Yolk was separated from the egg white and samples of both materials were stored at -70°C until assay for SA concentration.

### Determination of SA in plasma and eggs

Salicylate concentration was determined using high performance liquid chromatography (HPLC) method with ultraviolet (UV) detection, based on the method reported by Liu and Smith [11]. Samples were analysed using a Waters Alliance HPLC system with a Waters 2695 autosampler and Waters 2996 Photodiode Array (PDA) detector set at 203 nm (Waters, Milford, MA, USA). A 150×4.6mm i.d. reversed-phase column (5 μm Hypersil Gold aQ, Thermo Fisher Scientific, Waltham, MA, USA), attached to an appropriate guard column, was used. The mobile phase comprised 80% 0.01M NaH2PO4 (POCH, Gliwice, Poland) at pH 2.7 and 20% acetonitrile (J.T. Baker, Phillipsburg, NJ, USA). A gradient solvent program was run as follows: 0–14 min, 80/20; 14–17 min, 80/20:20/80; 17.1–20 min, 20/80:80/20 (0.01M NaH2PO4/acetonitrile). The flow rate was set at 1 ml/min. Plasma samples were deproteinized by mixing 250 ml of plasma with 750 ml acetonitrile in a 2 ml tube (Beckton Dickinson, NJ, USA). After this, the samples were vortexed for 1 min and centrifuged (32900×g, 30 min). The supernatant was transferred into clean tubes and evaporated under nitrogen. The residue was redissolved in water to the final volume of 250 ml and stored in -70°C until assay. Egg white and yolk samples were prepared by mixing 0.5 g of a respective egg fraction with 1 ml of 20% trichloroacetic acid solution in water (w/w) (Sigma, Germany). The resulting mixture was vortexed for 1 min and centrifuged (32900×g, 30 min). The supernatant was filtered and stored in -70°C until assay. After thawing, all samples were briefly vortexed, centrifuged (32900×g, 30 min) and 10 μl of the supernatant was injected into the instrument.

### Validation of the HPLC method

The specificity of the method was confirmed when no interfering peaks from endogenous compounds in different blank samples were observed at the same retention time as SA was present in the chromatograms of blank samples. Calibration curves were prepared by spiking blank samples of plasma, egg white or yolk with known concentrations of SA. Linearity of the analytical method was retained for a broad range of SA concentrations (0–300 μg/ml). Only one sample exceeded this concentration (331 μg/ml). To ensure highest quality of measurement, this sample was diluted, reassessed and the final value was calculated accordingly. The limit of detection (LOD), calculated as three times the ratio of the standard deviation of the peak area in the time of elution to the slope (LOD = 3×SD/Slope), was 0.13 μg/ml for plasma, 0.04 μg/ml for egg white and 0.03 μg/ml for yolk. The limit of quantification (LOQ), calculated as 10 times the ratio of the standard deviation to the slope (LOQ = 10×SD/Slope), was 0.39 μg/ml for plasma, 0.11 μg/g for egg white and 0.10 μg/g for yolk. The intra-assay coefficient of variation (CV) was 2.32% (at concentration 25 μg/ml), 2.64% (at concentration 6.25 μg/ml) and 4.03% (at concentration 1.56 μg/ml) and interassay CV was 3.23%, 4.84% and 8.96% at the respective concentrations.

### Analysis of data

For each individual, plasma SA concentration versus time data were subjected to non-compartmental pharmacokinetic analysis using Phoenix WinNonlin 6.3 (Certara, St. Louis, MO, USA). The linear trapezoidal approximation was used to calculate the area under the concentration-time curve (AUC) and the area under the first moment of the concentration-time curve (AUMC). The calculation included the time range from the first measurement to the last measurable drug concentration (AUC_last_, AUMC_last_), as well as the extrapolation to infinity (AUC_inf_, AUMC_inf_). The mean residence time (MRT_last_ and MRT_inf_), peak plasma concentration (C_max_), time when it was observed (t_max_), elimination half-life (T_1/2el_), total body clearence (Cl_B_) and volume of distribution at steady state (Vd_ss_) were determined. Plasma concentration at 16^th^ h after drug administration (C_16h_) was chosen as the representative point for the minimal concentration. In the lower dose group, at the 24^th^ h many individuals did not show detectable drug concentration in plasma which made the statistical analysis at that time point unreliable (it was calculated for the higher dose group as C_min_). Values of t_max_, C_max_, C_16h_ and C_min_ were presented as observed values. Degree of fluctuation (DF) was calculated as follows DF = 100% × (C_max_—C_min_)/C_avg_, where C_avg_ is the average concentration.

### Statistical analysis

All the data are presented as mean ± SD. To determine the relationship between the pharmacokinetic variables and the duration of SS administration, linear regression analysis was performed and Pearson’s correlation coefficient (r) was determined. Similarly, linear regression analysis was applied to investigate the relationship between the SA concentration in egg white and yolk, and the duration of treatment. To determine the statistical significance of the correlation coefficient, the Student’s t test was performed. In all cases, differences with P values > 0.05 were considered significant.

## Results

**Fig 1:**
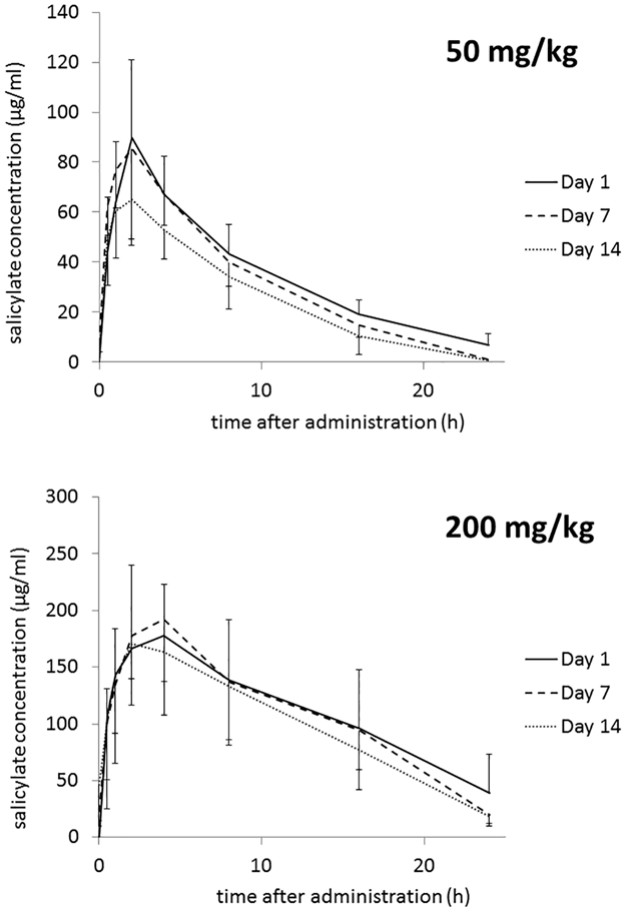
Plasma salicylate (SA) concentration time curves as determined on days 1, 7 and 14. Laying hens were treated daily with sodium salicylate (SS) at an oral dose of 50 mg/kg (upper panel) or 200 mg/kg (lower panel) for two weeks.

Plasma SA concentration time curves for both dose groups are shown in Fig 1. Curves obtained from three 24 h pharmacokinetic studies were overlaid to visualize changes that developed over 14 days. The time-dependent changes in pharmacokinetic variables are shown in Table 1. In the lower dose group (50 mg/kg), AUC, MRT, C_16h_, T_1/2el_ and Cl_B_ showed statistically significant decrease over time, whereas Cl_B_ significantly increased (statistical significance in Table 1 indicates a correlation between the duration of treatment and the change, either positive or negative, in a given variable). Values of Vd_ss_ and C_max_ remained unchanged. In the higher dose group (200 mg/kg), the slight decrease in most parameters did not reach statistical significance. Only T_1/2el_ was significantly decreased suggesting that similar mechanism as in the lower dose group was involved, although much less pronounced. Neither t_max_, nor DF changed in the course of the experiment.

**Table 1:** Pharmacokinetic variables (mean ± SD) of salicylate (SA) calculated from 3 subsequent experiments during two-week sodium salicylate (SS) oral administration in laying hens.

Daily dose	Variables	Unit	Day 1	Day 7	Day 14	r	Significance
			n = 8	n = 8	n = 6		
50 mg/kg	AUC_last_	mg×h/l	845 ± 168	736 ± 139	570 ± 165	-0.568	**
	AUC_inf_	mg×h/l	914 ± 201	828 ± 159	668 ± 202	-0.457	*
	MRT_last_	h	7.46 ± 0.76	5.82 ± 0.69	5.44 ± 1.12	-0.660	**
	MRT_inf_	h	9.35 ± 1.79	7.88 ± 1.17	6.77 ± 1.19^a^	-0.577	**
	C_max_	μg/ml	92.9 ± 28.5	91.8 ± 34.8	66.6 ± 14.0	-0.336	NS
	C_16h_	μg/ml	19.1 ± 5.7	14.6 ± 4.8	10.4 ± 7.4	-0.531	*
	T_1/2el_	h	6.13 ± 1.61	5.17 ± 0.80	4.35 ± 0.92^a^	-0.507	*
	Vd_ss_	l/kg	0.65 ± 0.24	0.61 ± 0.15	0.82 ± 0.16	0.320	NS
	Cl_B_	l/h×kg	0.07 ± 0.02	0.08 ± 0.02	0.12 ± 0.04	0.560	**
	t_max_	h	2.38 ± 1.06	1.63 ± 0.52	2.17 ± 0.98	-0.108	NS
	DF	%	171 ± 16	^b^	^b^	^b^	^b^
			n = 8	n = 8	n = 8		
200 mg/kg	AUC_last_	mg×h/l	2500 ± 1180	2654 ± 653	2396 ± 680	-0.053	NS
	AUC_inf_	mg×h/l	3141 ± 1777	2830 ± 723	2554 ± 687	-0.199	NS
	MRT_last_	h	8.74 ± 1.73	8.77 ± 1.08	8.52 ± 0.92	-0.072	NS
	MRT_inf_	h	13.49 ± 4.89	10.22 ± 1.56	10.11 ± 1.36	-0.391	NS
	C_max_	μg/ml	184.8 ± 59.9	203.4 ± 45.1	184.7 ± 54.2	-0.007	NS
	C_16h_	μg/ml	95.9 ± 51.6	94.2 ± 34.7	76.5 ± 34.7	-0.205	NS
	C_min_	μg/ml	38.7 ± 34.6	19.8 ± 9.9	18.2 ± 5.9	-0.377	NS
	T_1/2el_	h	8.62 ± 3.63	5.88 ± 1.26	5.82 ± 1.20	-0.419	*
	Vd_ss_	l/kg	1.24 ± 0.40	0.94 ± 0.40	1.03 ± 0.47	-0.178	NS
	Cl_B_	l/h×kg	0.12 ± 0.08	0.11 ± 0.03	0.12 ± 0.05	-0.001	NS
	t_max_	h	3.13 ± 1.25	2.75 ± 1.04	2.50 ± 1.31	-0.220	NS
	DF	%	140 ± 37	165 ± 13	163 ± 14	0.363	NS

For abbreviations see “material and methods” section. Statistical significance indicates a correlation between the duration of treatment (from day 1 to 14) and a change in the given variable as determined by linear regression analysis,

* P<0.05;

** P<0.01;

NS—non significant.

^a^ One individual showed values twice as high as in other individuals, thus was excluded as an outlier (in such case n = 5).

^b^ In these cases DF could not be calculated because SA was eliminated totally before the administration of the next dose.

**Fig 2:**
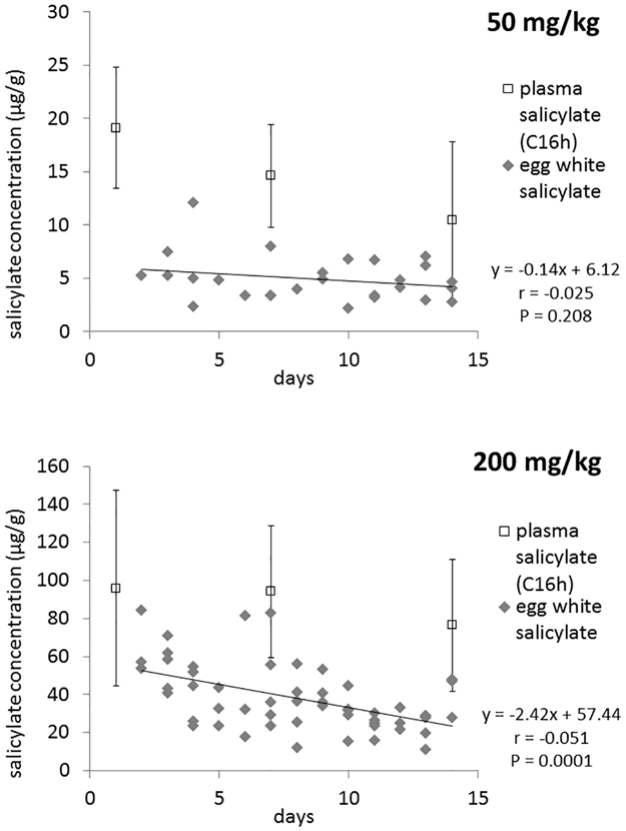
Salicylate (SA) concentrations in egg white of eggs collected from day 2 to 14. To show the relation with blood SA levels, plasma drug concentration at 16 h post administration (C_16h_) is included. Linear regression analysis was performed to investigate the statistical significance of the time-dependent changes in egg white SA.

**Fig 3:**
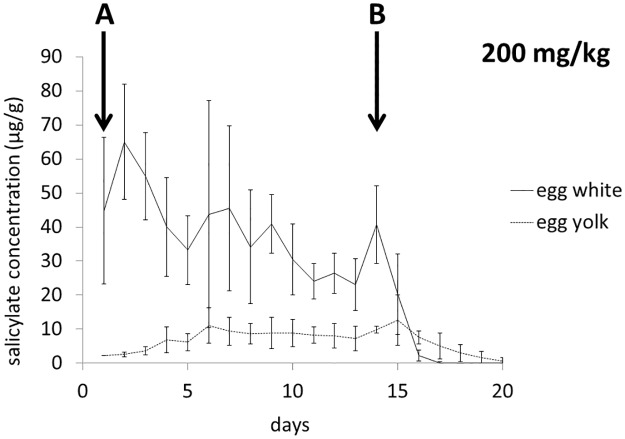
Time-dependent changes in egg white and yolk salicylate (SA) concentrations in the higher dose group (200 mg/kg). Letter (A) indicates the onset of daily sodium salicylate (SS) administration and letter (B) points the last SS dose.

Fig 2 shows the SA concentration in the egg white of eggs laid during the experiment (eggs laid on day 1 have been excluded from the regression analysis due to possible incomplete drug distribution). The results are projected on the SA concentrations in plasma as determined at the 16 h post administration (C_16h_). Interestingly, the time-dependent decrease in the drug concentration was observed only in the high dose group (the lower number of eggs in the low dose group was partially caused by experiment-independent factors like destruction by hens and loss during handling, thus should not be interpreted as the direct effect of drug administration). Fig 3 depicts the time-dependent changes in egg white and yolk SA concentrations in the higher dose group (no SA was found in yolks of the low dose group). It is apparent that much more SA is distributed to the egg white, whereas the distribution to the yolk is much lower and extended in time. After the administration was stopped, the egg white concentrations of SA decreased rapidly, whereas for yolk it took 6 days to eliminate the drug completely.

## Discussion

The social awareness of animal welfare and the necessity for pain management in farm animals have contributed to a greater need for the study of pharmacological aspects of analgesia in poultry [12]. However, for a veterinary practitioner the assessment of pain in birds is often a challenging task. Although more and more analgesics and anti-inflammatory drugs are available on the market, their dosage regimens are mainly based on pharmacokinetic studies of single administration only. In real life situations, these regimens may not always reflect the actual needs, especially if repeated or prolonged drug administration is performed.

The present study confirmed our previous findings that repeated administration of SS to chickens induces processes that lead to the gradual decrease in plasma drug concentration. However, this effect was much more pronounced in the group treated with the lower dose of SS (50 mg/kg) as compared to the higher dose group (200 mg/ml). In the lower dose group, AUC and MRT, which describe the extent of the drug’s presence in the body, significantly decreased. Lack of statistical importance of the decrease in C_max_ (which is a parameter more descriptive for the absorption phase of pharmacokinetic processes) may suggest that the observed decrease in AUC and MRT are mainly due to induced elimination. The decrease in the T_1/2el_ and C_16h_, and almost doubled Cl_B_ support this interpretation. The case of the higher dose group is more complex. Although the decreasing tendency was observed in most pharmacokinetic parameters studied, the statistical significance was found only for T_1/2el_. It is important to note that T_1/2el_ is calculated based on the drug concentration measurements taken in the second half of the experiment, thus is more representative for the elimination phase than other parameters which are calculated based on all measurements. In our opinion, the observed decrease in T_1/2el_ without statistically significant changes in AUC and MRT are indicative of accumulation processes playing a major role in the first week of the experiment (compare the C_max_ between Day 1 and 7). Thus, the seemingly constant SA kinetics in the higher dose group is in fact a dynamic transition from the initial drug accumulation to the subsequent induced elimination. This interpretation is further supported by the tendency of SA levels found in egg whites (Fig 2). Although the projected plasma drug concentration (C_16h_) does not show significant decrease, the concentration in egg white falls evidently. This indicates lower distribution of SA into egg white and supports the dynamic nature of changes in the drug’s pharmacokinetics during repeated administration. In the lower dose group, the decrease in egg white SA did not show statistical significance despite the significant decrease in plasma drug concentration. One possible explanation is that the mechanisms responsible for the effect observed in the higher dose group require larger doses of SA as a trigger. Another interesting observation was the difference in egg white and yolk SA concentration (Fig 3). This reflects the physicochemical nature of the drug and physiological difference in the formation of these two compartments. Salicylate is a weak acid characterized by high hydrophilicity and low polar surface area so it shows much higher permeation into the aqueous egg white as compared to much more lipophilic yolk. On the other hand, the much slower build-up of SA in yolk as well as the relatively long clearance reflect the time needed for yolk formation. It takes 9–10 d for an ovum to complete the rapid growth phase during which the majority of yolk volume is formed [13]. Since yolk is from this moment confined within membranes, SA distribution to this compartment is diminished. After ovulation, egg white is being formed and water with crystalloids are delivered in the process of “plumping” [13]. It is probably this latter phase in which the majority of SA enters the future egg white compartment. Since plumping takes place within about 5 hours preceding oviposition [14], egg white responds to plasma SA levels much faster than yolk does.

In our previous study on broilers [10], the decrease in plasma trough concentration was significant in groups treated with 200 and 400 mg/kg (the only doses applied). The minimal plasma levels of the drug decreased sharply after only 5 days of treatment, and by the 10^th^ day, most animals treated with the highest dose (400 mg/kg) showed concentrations significantly below 50 μg/ml which is considered the therapeutic level for this drug [7, 15, 16]. Trough concentrations in birds receiving the lower dose of 200 mg/kg never reached this level but decreased even further instead [10]. In laying hens used in the present study, the C_min_ was always below the therapeutic level of 50 μg/ml. On days 1, 7 and 14, in the low dose group (50 mg/kg) such concentration was sustained for 6.9, 6.5 and 4.5 h, whereas in the high dose group (200 mg/kg) for 22.4, 20.1 and 19.6 h. The clinical implications of this fact may probably include weaker anti-inflammatory and analgaesic effects, especially if SS is used at low dose for a longer time.

Although the European law does not permit the use of salicylates to treat laying hens at the moment, in many other countries ASA and SS are widely used for this purpose. There is, however, little data available on SA distribution into eggs. McDaniel *et al*. investigated the distribution of C^14^-labeled ASA into eggs of White Leghorns. Similar to our findings, they observed much higher levels of SA in the egg white as compared with the yolk [17]. They also found that eggs collected from hens administered with the dose of 0.05% (comparable to 50 mg/kg body weight) did not show detectable SA levels (<5 ppm). In the present study, a more sensitive method allowed for the detection of SA in the egg white from the low dose group (50 mg/kg) throughout the whole experiment. Salicylates are considered responsible for pseudo-allergic reactions in humans [18]. Since according to [19], 2.5% of European population is affected by SA intolerance, the control of residues in eggs is of particular importance. The exact mechanism responsible for the induced SA elimination is unknown. However, induced drug metabolism seems to be the most possible explanation [20]. Findings of Baert *et al*. (2004) indicate a broad inter-species variability in SA metabolism among birds. In case of *Galliformes*, most important metabolites include gentisic acid and ornithine conjugate [21]. In humans, it was found that both cytochrome P4502E1 and 3A4 are involved in SA hydroxylation to gentisic acid [22]. In rats, SA was found to be an inducer of P4502E1 [23]. Analogic isoform, as determined by the ability to metabolize aniline, was found to be very active in chicken liver [24]. Unfortunately, gentisic acid and especially ornithine conjugate were not detectable by the applied analytical method, thus it is impossible to assess which of these pathways may have been involved in the proposed metabolic induction.

It is concluded that repeated administration of SS causes induced SA elimination. This effect is observed in both broiler chickens [10] and laying hens. It may, however, differ quantitatively depending on the production type of a chicken. Salicylate is distributed into eggs, particularly to the egg white. During repeated administration, this distribution decreases significantly, especially if higher doses are applied. The observed phenomenon may have clinical implications, e.g. decreased pharmacological efficacy, during prolonged SA treatment in chickens. It is important to note that the observed effect may as well apply to other drugs of different chemical formula and activity (e.g. antimicrobials). Since the dosage regimens are often designed based on pharmacokinetic analysis of a single administration, it may be well advised to reassess some drugs in terms of their pharmacokinetics after repeated administration. This may be of particular interest if clinical effects of a given drug seem to decline with time.
